# Establishment and evaluation of a nomogram for in-hospital new-onset atrial fibrillation after percutaneous coronary intervention for acute myocardial infarction

**DOI:** 10.3389/fcvm.2024.1370290

**Published:** 2024-03-18

**Authors:** Junjie Tu, Ziheng Ye, Yuren Cao, Mingming Xu, Shen Wang

**Affiliations:** ^1^ The Second School of Clinical Medicine, Zhejiang Chinese Medical University, Hangzhou, China; ^2^ Department of Cardiovascular Medicine, Zhejiang Provincial People's Hospital (Affiliated People's Hospital, Hangzhou Medical College), Hangzhou, China; ^3^ Department of Cardiovascular Medicine, Zhejiang Greentown Cardiovascular Hospital, Hangzhou, China

**Keywords:** acute myocardial infarction, new-onset atrial fibrillation, percutaneous coronary intervention, risk prediction model, nomogram

## Abstract

**Background:**

New-onset atrial fibrillation (NOAF) is prognostic in acute myocardial infarction (AMI). The timely identification of high-risk patients is essential for clinicians to improve patient prognosis.

**Methods:**

A total of 333 AMI patients were collected who underwent percutaneous coronary intervention (PCI) at Zhejiang Provincial People's Hospital between October 2019 and October 2020. Least absolute shrinkage and selection operator regression (Lasso) and multivariate logistic regression analysis were applied to pick out independent risk factors. Secondly, the variables identified were utilized to establish a predicted model and then internally validated by 10-fold cross-validation. The discrimination, calibration, and clinical usefulness of the prediction model were evaluated using the receiver operating characteristic (ROC) curve, calibration curve, Hosmer-Lemeshow test decision curve analyses, and clinical impact curve.

**Result:**

Overall, 47 patients (14.1%) developed NOAF. Four variables, including left atrial dimension, body mass index (BMI), CHA_2_DS_2_-VASc score, and prognostic nutritional index, were selected to construct a nomogram. Its area under the curve is 0.829, and internal validation by 10-fold cross-folding indicated a mean area under the curve is 0.818. The model demonstrated good calibration according to the Hosmer-Lemeshow test (*P* = 0.199) and the calibration curve. It showed satisfactory clinical practicability in the decision curve analyses and clinical impact curve.

**Conclusion:**

This study established a simple and efficient nomogram prediction model to assess the risk of NOAF in patients with AMI who underwent PCI. This model could assist clinicians in promptly identifying high-risk patients and making better clinical decisions based on risk stratification.

## Introduction

Despite the rapid and widespread development of antithrombotic and reperfusion therapies, acute myocardial infarction (AMI) continues to be a leading cause of global mortality (1). The occurrence of New-onset atrial fibrillation (NOAF) is a frequently observed arrhythmic complication in patients with AMI and has been identified as an independent risk factor for adverse cardiovascular events, with reported prevalence rates ranging from 2.3% to 23% (2–4). The presence of atrial fibrillation (AF), whether transient or persistent, can result in prolonged hospitalization and increased short- and long-term cardiovascular adverse events and mortality (5, 6). In cases of AMI complicated by NOAF post-percutaneous coronary intervention (PCI), careful consideration of antiplatelet and anticoagulant treatment options was crucial. It could mitigate heightened risks of ischemic stroke, recurrent coronary events, stent thrombosis, and bleeding. Moreover, the recurrence rate of AF is significantly higher in patients with transient NOAF or those who experience conversion to sinus rhythm during hospitalization (7). This particular subgroup of patients may have been overlooked for anticoagulation treatment due to issues related to poor adherence and inadequate follow-up electrocardiogram monitoring. Additionally, several studies have demonstrated the potential of specific medications to inhibit the incidence of NOAF (8, 9). Diverse studies reported a wide range of incidence rates. In practical clinical management, there was a lack of cost-effective and easily implementable methods for real-time monitoring of transient NOAF in practical clinical settings. Timely identifying high-risk patients during their hospital stay may facilitate the application of targeted aggressive drug therapies to mitigate NOAF occurrence or enhance the prognosis of these patients through more comprehensive clinical management programs.

The nomogram serves as a simple and practical predictive tool that combines significant predictors to visually forecast clinical events and disease prognosis. The risk factors of NOAF following AMI have been extensively investigated in numerous studies, yet only a few have developed prediction tools suitable for clinical application. Therefore, this study aimed to construct a concise and effective nomogram model that accurately predicts the risk of NOAF in hospitalized patients.

## Materials and methods

### Study population

This study retrospectively and consecutively enrolled AMI patients who underwent PCI at Zhejiang Provincial People's Hospital from October 2019 to October 2020. Their diagnosis adhered to the European Society of Cardiology/American College of Cardiology consensus criteria (10). Patients with a history of AF were excluded. Exclusion criteria included: (1) severe valvular disease, congenital heart disease; (2) severe infection, liver and kidney failure, and malignant tumors; (3) death during hospitalization; (4) insufficient clinical data. We recruited 383 patients and excluded 50 patients according to the above exclusion criteria: 13 patients with severe renal failure, 5 patients with severe infections, 15 patients with malignant tumors, 6 patients who died during hospitalization, and 11 patients with insufficient clinical data. Finally, 333 patients were included in this study. This study was conducted in accordance with the Declaration of Helsinki, ethical approval was obtained from the Zhejiang Provincial People's Hospital ethics committee. As we had a retrospective study design, written informed consent was waived with the permission of the ethics committee.

### Clinical data collection

The patient's demographic information collected through the electronic medical record system includes age, gender, height, weight, body mass index (BMI), smoking history, drinking history, admission conditions [systolic blood pressure (SBP), and diastolic blood pressure (DBP), heart rate (HR), Killip classification, type of acute myocardial infarction], presence of comorbidities (hypertension, stroke, diabetes, coronary heart disease), Long-term administration of medications before admission [angiotensin-converting enzyme inhibitor or angiotensin receptor blocker (ACEI or ARB), beta-blocker, statin]. Laboratory test results within 24 h of admission include: serum albumin, total cholesterol (TC), triglycerides (TG),high-density lipoprotein cholesterol (HDL-C), low-density lipoprotein cholesterol (LDL-C), uric acid, serum creatinine(Scr), estimated glomerular filtration rate (eGFR), white blood cell count(WBC), lymphocyte count, monocyte count, red blood cell count(RBC), hemoglobin, neutrophil count, platelet count (Plt), high-sensitivity C reactive protein (Hs-CRP), B type natriuretic peptide (BNP), and cardiac troponin I levels (cTnI). Transthoracic echocardiography results within 24 h of admission: left atrial dimension (LAD), left ventricular ejection fraction(LVEF). Coronary artery stenosis is determined based on the results of coronary angiography, the stenosis degree of a certain coronary artery is ≥50% (including the left main artery, left anterior descending artery, left circumflex artery, and right coronary artery). Calculate the CHA_2_DS_2_-VASc score based on the above conditions. The prognostic nutritional index (PNI) is calculated from the formula: 10 × serum albumin (g/dl) + 0.005 × total lymphocyte count (mm^3^) (11).

### Clinical follow-up and endpoints

A 12-lead electrocardiogram is routinely performed on admission, post-PCI, and before discharge. Additionally, a 24-hour dynamic electrocardiogram is completed postoperatively. Furthermore, continuous telemetry electrocardiograms are routinely performed to monitor the patient's heart rhythm. When the nurse identifies a suspicious NOAF through the computer cloud platform, a complete bedside 12-lead electrocardiogram is conducted. The follow-up endpoint is discharge or the occurrence of NOAF during hospitalization. The diagnosis of NOAF is in line with the guideline consensus, which is defined as an absolutely irregular RR interval, no discernible *P* waves, and an episode lasting ≥30 s (12).

### Model establishment, validation and evaluation

Considering the large number of variables included in this study, 45 clinical variables were downscaled using Least absolute shrinkage and selection operator (LASSO) regression to screen out the characteristic variables with non-zero coefficients. Subsequently, the characteristic variables were further analyzed by multivariate logistic regression to select the independent predictors of in-hospital NOAF after PCI in patients with AMI. We constructed a nomogram utilizing independent predictors identified (*P* < 0.05) to predict the risk of NOAF. The predictive efficacy of the model was evaluated by plotting a receiver operating characteristic (ROC) curve. Then we performed internal validation of the model through the 10-fold cross-folding method. The Hosmer-Lemeshow(H-L) test was utilized to measure the fit of this model, and the calibration curve was drawn by resampling 1,000 times using the Bootstrap method to evaluate the degree of calibration of the model. Finally, we evaluated the clinical utility of the model using decision curve analyses (DCA) and Clinical Impact Curve (CIC).

### Statistical analysis

The normality of continuous variables was assessed using the Kolmogorov-Smrinov test combined with the Q-Q plot and was expressed as mean ± SD or median M (P25, P75), according to whether it conformed to the normal distribution. Categorical variables are expressed as counts and percentages (%). The Student's *t*-test was used for normal continuous variables, and the Mann-Whitney *U*-test was used for non-normal variables. Categorical variables were subjected to a Chi-square test. A two-sided *P* < 0.05 was considered a statistically significant difference. Independent predictors were identified through LASSO regression and multivariate logistic regression. A nomogram model was developed using the aforementioned predictors. The model was evaluated by area under the ROC curve, calibration curve, H-L test, DCA, and CIC. Internal validation was performed by the 10-fold cross-folding method. All statistical analyses were performed using R software (version 4.1.1, https://www.r-project.org/).

## Result

### Patient characteristics

Our cohort finally included 47 (14.1%) patients in the NOAF group and 286 (85.9%) patients in the sinus rhythm (SR) group. The baseline characteristics of 2 groups are shown in Table 1. Analysis revealed that age, weight, BMI, CHA_2_DS_2_-VASc score, Killp classification, serum albumin, TG, Scr, eGFR, lymphocyte count, RBC, hemoglobin, Hs-CRP, BNP, PNI, LAD, LVEF, and left circumflex artery stenosis were statistically significant variables.

**Table 1:** Patient baseline characteristics.

Variables	SR (*n* = 286)	NOAF (*n* = 47)	*P*-value
Basic characteristics			
Age(year)	61.24 ± 12.03	70.3 ± 11.18	<0.001
Male (*n*, %)	232 (81.1)	37 (78.7)	0.699
Weight (kg)	68.57 ± 11.22	61.9 ± 11.4	<0.001
Height (cm)	166.71 ± 7.03	166.45 ± 7.37	0.813
BMI (kg/m^2^)	24.6 ± 3.3	22.21 ± 2.9	<0.001
SBP on admission (mmHg)	141.01 ± 25.22	133.7 ± 28.65	0.072
DBP on admission (mmHg)	84.86 ± 17.93	79.79 ± 14.38	0.066
HR on admission (bmp)	80.81 ± 15.89	84.96 ± 19.24	0.109
Smoking history (*n*, %)	127 (44.4)	23 (48.9)	0.563
Drinking history (*n*, %)	97 (33.9)	14 (29.8)	0.578
STEMI(n,%)	137 (47.9)	27 (57.4)	0.225
CHA_2_DS_2_-VASc score	2.93 ± 1.54	4.28 ± 1.83	<0.001
Killp class (*n*, %)			<0.001
I			
II			
III			
IV			
Medications on admission			
ACEI/ARB (*n*, %)	68 (23.8)	10 (21.3)	0.708
Beta-blocker (*n*, %)	14 (4.9)	2 (4.3)	1.000
Statin (*n*, %)	27 (9.4)	2 (4.3)	0.374
Complications			
Hypertension (*n*, %)	173 (60.5)	31 (66)	0.476
Diabetes (*n*, %)	72 (25.2)	17 (36.2)	0.114
Stroke/TIA (*n*,%)	19 (6.6)	5 (10.6)	0.498
Coronary heart disease (*n*,%)	35 (12.2)	6 (12.8)	0.919
Laboratory results			
Serum albumin (g/dl)	37.32 ± 3.38	34.56 ± 4.93	<0.001
TG(mmol/L)	1.38 (1.03−2.17)	1.07 (0.86–1.52)	0.002
TC(mmol/L)	4.67 ± 1.24	4.35 ± 1.5	0.116
LDL_C(mmol/L)	85.88 ± 22.61	93.03 ± 24.08	0.380
HDL_C(mmol/L)	0.97 (0.83–1.14)	0.98 (0.82–1.12)	0.895
Uric acid (umol/L)	358.39 ± 99.26	368.3 ± 110.32	0.533
Scr (umol/L)	81.5 (72.73–93.05)	94.3 (80.9–101.7)	0.004
eGFR (ml/min × 1.73 m2)	78.15 ± 22.2	69.26 ± 22.56	0.012
WBC(10^9^/L)	8.81 (7.09–11.44)	9.7 (6.81–11.15)	0.652
Lymphocyte count (10^9^/L)	1.6 (1.1–2.21)	1.1 (0.8–1.5)	<0.001
Monocyte count (10^9^/L)	0.5 (0.35–0.6)	0.5 (0.34–0.6)	0.486
Neutrophil count (10^9^/L)	6.12 (4.7–8.83)	7.5 (4.3–9.3)	0.552
RBC(10^12^/L)	4.58 ± 0.6	4.3 ± 0.63	0.003
Hemoglobin (g/L)	142.79 ± 19.22	132.79 ± 21.58	0.001
Plt(10^9^/L)	193 (159.75–233)	185 (137–251)	0.347
Hs.CRP(mg/L)	3.1 (1.3–9.45)	8.6 (2.8–39.8)	<0.001
BNP(pg/ml)	113.25 (50.4–245.23)	311.8 (161.2–1,012.5)	<0.001
cTnI (ug/L)	1.51 (0.19–9.08)	1.79 (0.46–12.49)	0.609
PNI	45.9 (42.08–49.75)	40.9 (37.1–44.8)	<0.001
Echocardiography results			
LAD(mm)	37.47 ± 4.66	39.87 ± 5.45	0.002
LVEF (%)	59 (55–63)	53 (44–59)	<0.001
Coronary artery stenosis > 50%,(*n*, %)			
Left main artery	22 (7.7)	3 (6.4)	0.986
Left anterior descending artery	218 (76.8)	41 (87.2)	0.092
Left circumflex artery	142 (49.7)	32 (68.1)	0.019
Right coronary artery	164(57.3)	29(61.7)	0.575

BMI, body mass index; SBP, systolic blood pressure; DBP, diastolic blood pressure; HR, heart rate; STEMI, ST segment elevation myocardial infarction; ACEI/ARB, angiotensin-converting enzyme inhibitor or angiotensin receptor blocker; TG, triglycerides; TC, total cholesterol; LDL-C, low-density lipo-protein cholesterol; HDL-C, high-density lipoprotein cholesterol; Scr, serum creatinine; eGFR, estimated glomerular filtration rate; WBC, white blood cell count; RBC, red blood cell count; Plt, platelet count; Hs-CRP, high-sensitivity C reactive protein; BNP, B type natriuretic peptide; cTnI, cardiac troponin I levels; PNI, prognostic nutritional index; LAD, left atrial dimension; LVEF, left ventricular ejection fraction.

### Feature selection and establishment of nomogram

**Figure 1:**
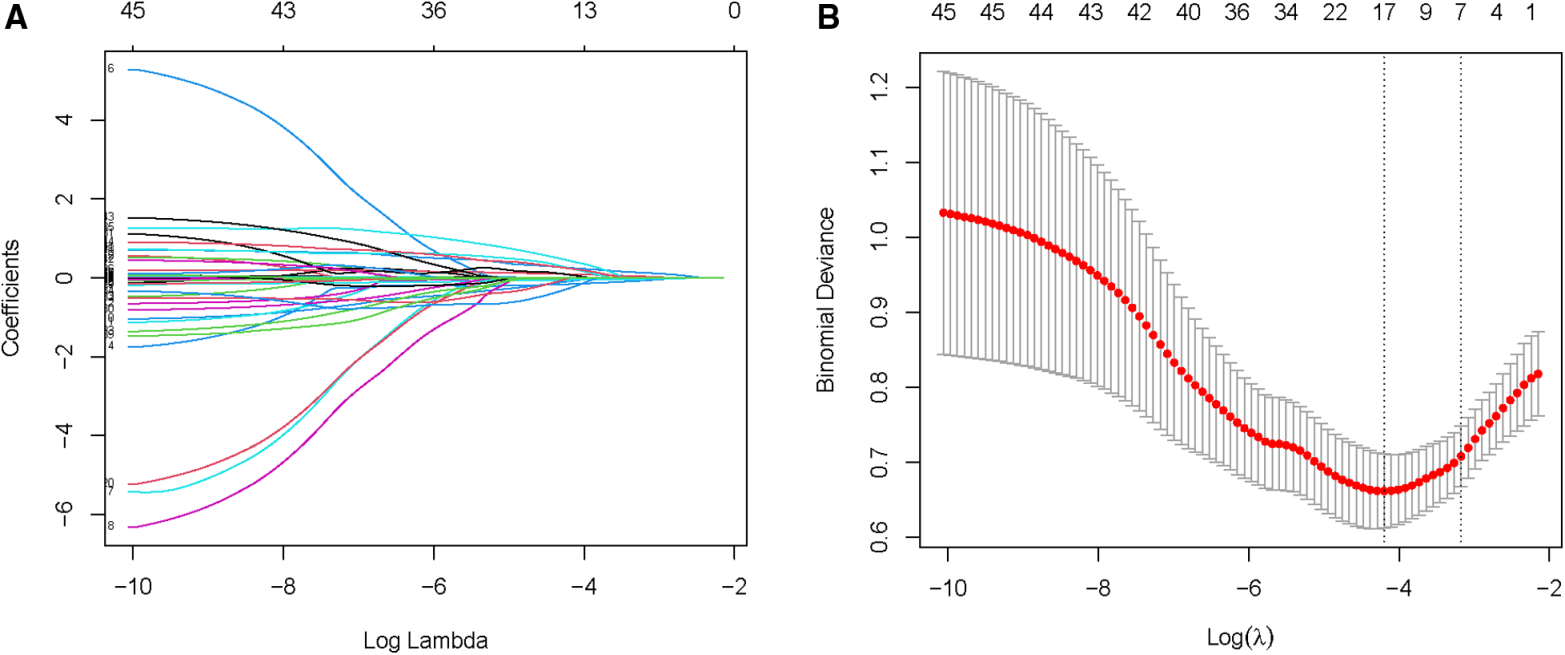
Selection of NOAF risk factors using the LASSO regression method. (**A**) LASSO coefficient profiles of the 45 texture features. A coefficient profile plot is produced against the log (*λ*) sequence. (**B**) LASSO regression model 10-fold cross-validation plot. Draw a vertical line at the optimum with the minimum criterion and 1se of the minimum criterion. When *λ* = 0.040, we get 7 variables for further analysis.

**Figure 2:**
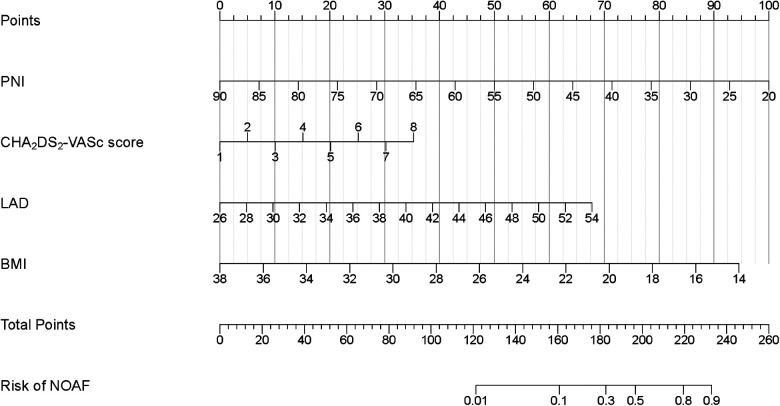
Developed in-hospital NOAF prediction nomogram in patients after PCI with AMI. Each clinical variable has a certain number of points. The sum of points of each variable was related to the probability of NOAF. For each covariate, please draw a vertical line upwards and note down the corresponding points. This is repeated for each covariate ending with a total score that corresponds to a predicted risk of NOAF at the bottom of the nomogram. PCI, percutaneous coronary intervention; AMI, acute myocardial infarction; NOAF, new-onset atrial fibrillation; BMI, body mass index; PNI, prognostic nutritional index; LAD, left atrial dimension.

Due to the inclusion of numerous variables, LASSO regression was used for dimensionality reduction and screening. Seven key variables were determined to be important for NOAF: BMI, CHA_2_DS_2_-VASc score, Hs-CRP, BNP, PNI, LA, and LVEF (Figures 1A,B). Subsequently, we conducted a multivariate logistic regression analysis to recognize independent risk factors for NOAF. The results indicated that the four variables of BMI, CHA_2_DS_2_-VASc score, PNI, and LA were statistically significant (*P* < 0.05) as shown in Table 2. Based on these variables, a nomogram was constructed (Figure 2).

**Table 2:** Multivariate logistics regression for predicting in-hospital NOAF after PCI in patients with AMI.

Variables	Coef.	OR	95%CI	*P*-value
BMI (kg/m^2^)	−0.222	0.800	0.694–0.915	0.002
Hs.CRP (mg/L)	0.007	1.007	0.999–1.016	0.080
BNP (pg/ml)	0.001	1.000	0.999–1.235	0.659
CHA_2_DS_2−_VASc score	0.256	1.291	1.021–1.639	0.034
LAD (mm)	0.133	1.142	1.060–1.235	<0.001
LVEF (%)	−0.036	0.965	0.927–1.016	0.078
PNI	−0.070	0.932	0.870–0.992	0.037

BMI, body mass index; Hs-CRP, high-sensitivity C reactive protein; BNP, B type natriuretic peptide; LAD, left atrial dimension; LVEF, left ventricular ejection fraction; PNI, prognostic nutritional index.

### Nomogram evaluation and validation

**Figure 3:**
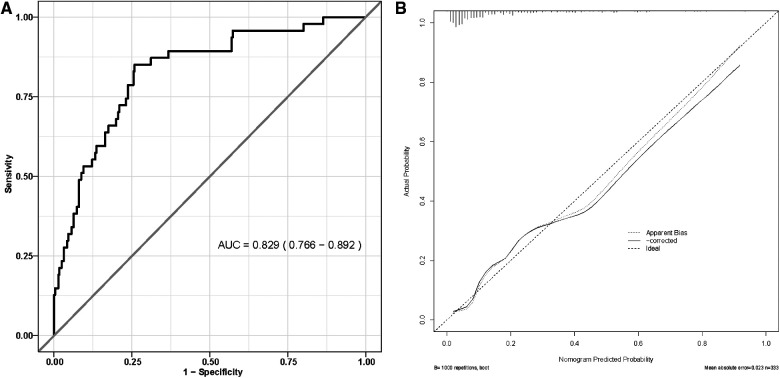
(**A**) Receiver operating characteristic curve of nomogram model; (**B**) the calibration curve for nomogram model.

We generated a ROC curve to evaluate the model's distinguishing ability, AUC = 0.829 (95% CI: 0.766–0.892) (Figure 3A). Internal validation was performed using the 10-fold cross-folding method with 1,000 iterations, revealing an average AUC of 0.818, indicative of the model's robust discriminative ability. Furthermore, the calibration curve (Figure 3B) and H-L test (*P* = 0.199) demonstrated a high degree of consistency between the predicted survival probability and actual survival proportion.

### Clinical application of the model

**Figure 4:**
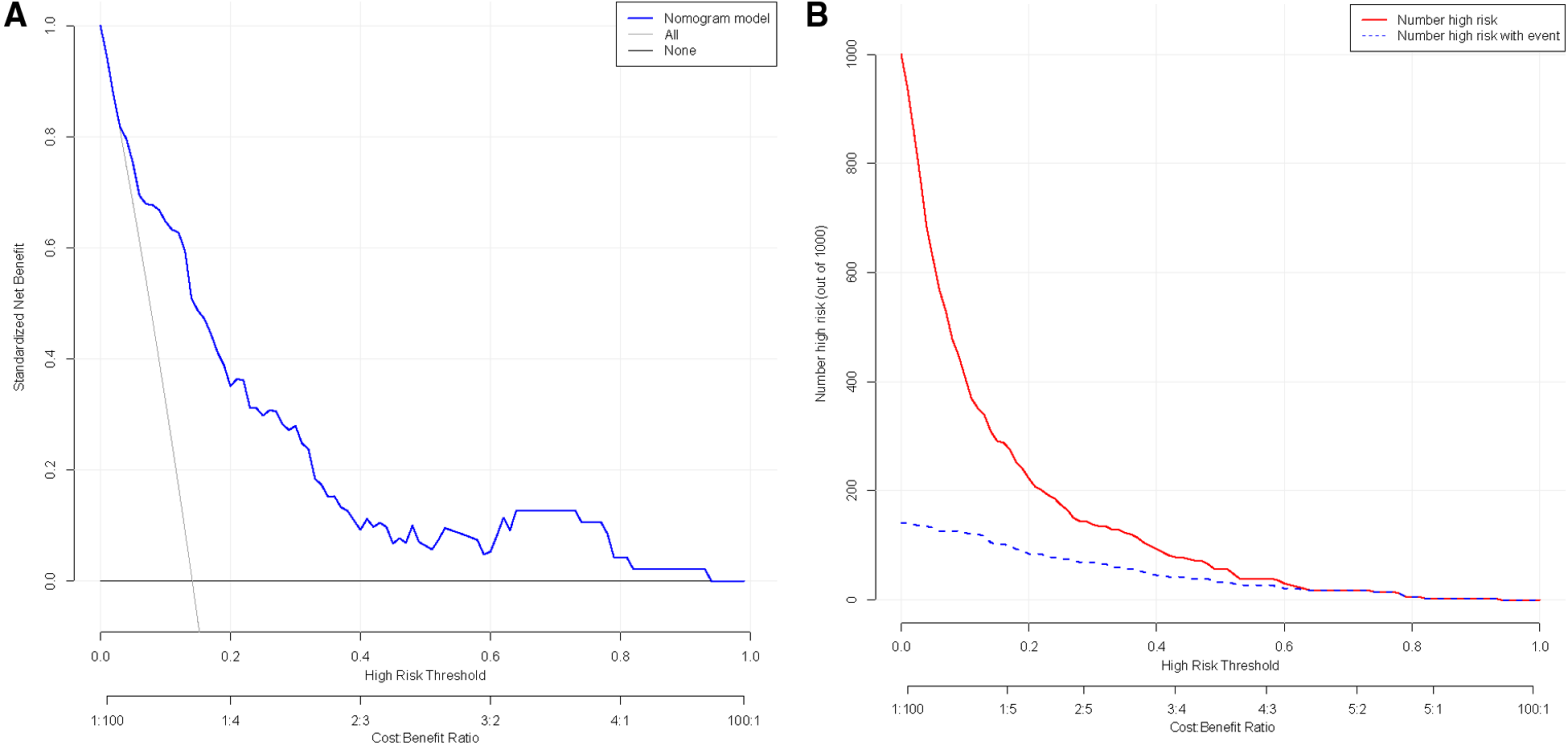
(**A**) Clinical decision curve analysis for nomogram models; (**B**) analysis of clinical impact curve for nomogram model.

The clinical utility was assessed by constructing DCA and CIC. In the DCA, two lines are used to represent extreme scenarios. Specifically, the horizontal line indicates no intervention for all patients, resulting in no net clinical benefit. On the other hand, the left diagonal line represents the benefit when all patients receive the intervention. The blue curve shows a scenario where the intervention is given to patients based on the model. Consequently, the range of clinical benefit obtained from this model lies below the blue curve and beyond the horizontal and left diagonal lines. The net clinical benefit found in the model ranges from 1% to 93%. (Figure 4A) The CIC used the model to simulate predicted clinical benefit for 1,000 patients. The red curve shows the number of individuals predicted by the model to experience an outcome event at different thresholds, while the blue curve shows the actual occurrence of the outcome event. As shown, the two curves demonstrate favorable concordance within a specific range. (Figure 4B) In summary, the model has demonstrated good clinical utility.

## Discussion

In this study, we retrospectively analyzed the data of 333 AMI patients who underwent PCI at Zhejiang Provincial People's Hospital. Lasso regression and multivariate logistic regression were employed to identify four independent risk factors: PNI, BMI, CHA_2_DS_2_-VASc score, and LAD. Subsequently, a nomogram prediction model was developed. The model demonstrated excellent discriminative ability for predicting NOAF during hospitalization (AUC = 0.829) and was internally validated using 10-fold cross-folding (average AUC = 0.818). In addition, the calibration curve and H-L test indicated good calibration of this model. Finally, the DCA and CIC confirmed its significant clinical utility.

The CHA_2_DS_2_-VASc score was designed to foresee the danger of thromboembolism in AF patients and was recommended by guidelines for widespread clinical use. It comprises hypertension, diabetes, age, congestive heart failure, prior stroke/ transient ischemic attack, female gender, and vascular disease (13). Interestingly, these indicators are also recognized risks for AF, which to some extent reflect the long-term left atrial structure and electrical remodeling, as well as hemodynamic disorders in the acute phase of AMI. In a large sample study from Israel, the CHA_2_DS_2_-VASc score was found to be useful for risk assessment of NOAF in the general population with an AUC = 0.744. The hazard ratio for AF was 1.57 (95% CI 1.56–1.58) for each 1-point increase (14). In addition, a study in an AMI cohort found that the CHA_2_DS_2_-VASc score could assess the long-term risk of NOAF, whether the patient underwent surgery (PCI or coronary artery bypass grafting) (15). However, other studies reported that it didn't have good identification ability in ST-segment elevation myocardial infarction (STEMI) patient cohorts, with AUC≈0.67 (16, 17). In any case, this study identified it as a robust predictive factor through LASSO regression and multivariate logistic regression, which is consistent with the above research. LAD assessed by transthoracic echocardiography is the easiest clinical indicator to evaluate left atrial structural remodeling and serves as a potent predictor of the occurrence and recurrence of AF (18, 19).

Recently, an increasing number of studies have found that malnutrition was closely associated with poor prognosis in patients with cardiovascular disease (20–22). Data from Europe and the Americas showed that up to one-third of hospitalized patients were at risk of malnutrition or were malnourished upon admission, especially in acutely ill patients. Nutritional status in these cases was further worsened due to disease progression or side effects of medications (23). Similarly, a study from the Chinese CIN and American MIMIC-III database reported that one-third of AMI patients were also at high risk of malnutrition (24). Studies have reported that malnutrition could advance the happening of NOAF in AMI patients (25, 26). Following adjustment for confounding factors, this study found that nutritional status assessed by BMI and PNI was an independent risk factor for NOAF. Xiangrong, X and Yan, C observed that malnutrition assessed by PNI promoted the occurrence of NOAF in a cohort of ST-segment elevation myocardial infarction patients similarly (27). PNI is a simple nutritional assessment tool that reflects inflammation and immunity through peripheral albumin and lymphocyte composition. Serum albumin has anti-inflammatory properties. Hypoalbuminemia promotes the inflammatory response and exacerbates oxidative stress, thereby leading to the occurrence of NOAF (28). Meta-analyses and Mendelian randomization studies have confirmed that there was an observably negative linear relationship between serum albumin and the occurrence of AF (29, 30). Reduced lymphocyte counts might lead to immunosuppression in patients and to some extent reflected activation of the neurohormonal system and physiological stress (31). Disturbances of the autonomic neurohormonal system are one of the acknowledged mechanisms for the development and maintenance of AF (13). A large cohort study from an American community reported that lymphocyte count was negatively associated with the risk of AF (32). BMI is the most widely used measure of patient obesity and a traditional risk factor for cardiovascular disease. However, there is an “obesity paradox” in certain circumstances, such as heart failure, stable coronary artery disease, AF, and acute coronary syndrome (33–35). that is, obese patients had a better prognosis in these specific populations. A recent study has demonstrated that this paradox is similarly present in a population with NOAF after AMI (36). Furthermore, a meta-analysis of the Acute Coronary Syndrome cohort, encompassing 24 studies and involving 585,919 participants, revealed a significant and negative association between BMI and prognostic outcomes, including cardiogenic shock, mortality, and in-hospital complications in this cohort (37). We hypothesize that the “obesity paradox” may be due to selection bias. Obese patients are relatively young, yet patients with cardiovascular disease are more likely to be found in the elderly (38). Secondly, patients with higher BMI have more adipose tissue, leading to the production of adiponectin with anti-inflammatory, anti-atherosclerotic, and antioxidant properties. This can help to reduce the incidence of NOAF by attenuating myocardial damage from AMI (39). Nevertheless, NOAF occurs when patients with AMI are at relative risk of malnutrition.

In the era of widespread PCI adoption, the mortality rate in the acute phase of patients with AMI has been significantly improved. However, when patients develop NOAF, the patient's prognosis is often further aggravated (40). Certain studies have reported that transient NOAF accounts for the majority (41). Such patients have a greater risk of recurrence of atrial fibrillation and may be ignored due to poor compliance and inadequate follow-up management, potentially resulting in embolic events; When identified during hospitalization, physicians confront decisions regarding the regimen and timing of antiplatelet and anticoagulant therapy, balancing embolic and bleeding events. We developed a novel prediction model specifically designed to predict the occurrence of NOAF during hospitalization in patients with AMI. This model will help clinicians to risk-stratify AMI patients and make more reasonable treatment and follow-up decisions.

## Limitation

This study has the following limitations: First, this study is a single-center retrospective study, and there is selection bias in cases. Secondly, due to actual circumstances, we may not have included some risk factors that affect NOAF and may play a more important role. In addition, the sample size of this study is limited, and only internal validation was performed to prevent model overfitting, and no external validation of the model was performed, which may lead to the weakening of the generalizability and representativeness of our research results. Therefore, a large-sample, multicenter prospective study is required to confirm our results.

## Conclusion

In conclusion, based on the independent risk factors, the established prediction model has good discrimination and clinical utility. The application of this model will help identify high-risk individuals with internal NOAF after AMI, facilitating clinical decision-making and ultimately enhancing patient prognosis.

## Data Availability

The raw data supporting the conclusions of this article will be made available by the authors, without undue reservation.
